# Integration of Methylation and Gene Expression Deciphered Candidate Biomarkers DAB2IP and SMYD3 in Delayed Encephalopathy After Carbon Monoxide Poisoning

**DOI:** 10.1111/cns.70270

**Published:** 2025-02-15

**Authors:** Hongyi Yan, Ding Yuan, Yan Zhang, Haihua Luo, Pinpin Jiang, Yapeng Zhang, Yue Wu, Linlin Hou, Yue Cheng, Fang Yang, Yuqi Du, Huanzhou Zhu, Linshuang Zhao, Yi Li, Yong Jiang, Yanxia Gao

**Affiliations:** ^1^ Department of Emergency Medicine The First Affiliated Hospital of Zhengzhou University, Medical Key Laboratory of Poisoning Diseases of Henan Province Zhengzhou China; ^2^ State Key Laboratory of Antiviral Drugs, Henan Key Laboratory of Critical Care Medicine, Henan International Joint Laboratory of Infection and Immunology, Department of Emergency Medicine The First Affiliated Hospital, Zhengzhou University Zhengzhou China; ^3^ Institute of Infection and Immunity, Henan Academy of Innovations in Medical Science Zhengzhou China; ^4^ School of Pharmacy Fudan University Shanghai China; ^5^ Emergency Department, State Key Laboratory of Complex Severe and Rare Diseases Peking Union Medical College Hospital, Peking Union Medical College, Chinese Academy of Medical Sciences Beijing China

**Keywords:** delayed encephalopathy after carbon monoxide poisoning, DNA methylation, epigenetic biomarker, pyrosequencing, RNA sequencing

## Abstract

**Aims:**

The objective of this study is to explore the regulatory role of DNA methylation in delayed encephalopathy after carbon monoxide poisoning (DEACMP) and to identify candidate epigenetic biomarkers.

**Methods:**

In this study, multi‐omics analyses such as methylomics, transcriptomics, pyrophosphate sequencing, qRT‐PCR, immunohistochemistry, and western blotting were utilized to investigate the role of epigenetic regulation and altered gene expression in the pathogenesis of DEACMP.

**Results:**

Using integrated analysis, we identified 168 differentially methylated CpGs sites, 334 differentially expressed genes, and two differentially methylated and differentially expressed genes (DAB2IP and SMYD3) in the DEACMP group. The pyrosequencing results further revealed hypomethylation of DAB2IP and hypermethylation of SMYD3. Moreover, we verified the upregulation of DAB2IP expression accompanied by the downregulation of SMYD3 expression in the DEACMP rats model.

**Conclusion:**

This study, based on dysregulated DNA methylation and gene expression profiles, identified and validated two DEACMP‐related genes (DAB2IP and SMYD3) that could serve as epigenetic biomarkers and potential therapeutic targets for DEACMP.

## Graphical abstract

By integrating DNA methylation and gene expression from patients with delayed encephalopathy after carbon monoxide poisoning (DEACMP), this study identified DAB2IP and SMYD3 as candidate genes potentially involved in DEACMP pathogenesis. Additionally, further evidence for their potential as DEACMP targets was provided through pyrosequencing and DEACMP rat model.

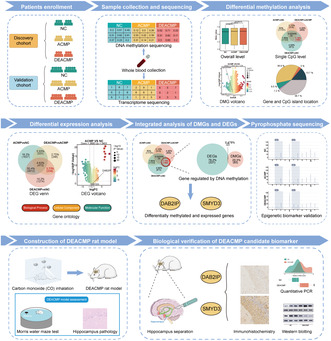

## 1 Introduction

Carbon monoxide (CO) is a major contributor to poisoning‐related injuries and deaths [1, 2]. Annually, approximately 50,000–100,000 individuals with CO poisoning visit emergency departments in the United States [3]. Up to 40% of patients with CO poisoning may develop delayed encephalopathy after acute carbon monoxide poisoning (DEACMP) within the 2–60 days latent phase. Despite combination therapies based on hyperbaric oxygen treatment, 50% of these patients experience cognitive, neurological, or neurobehavioral deficits [4]. From a genetic perspective, several genome‐wide association studies (GWAS) have identified single‐nucleotide polymorphisms (SNPs) in genes such as NSE and NRXN3, which may increase susceptibility to DEACMP [5, 6]. DEACMP is mainly caused by a series of pathophysiological changes induced by CO as an environmental factor. Epigenetic modifications can indicate a correlation between environmental risk factors and disease phenotypes, which may be valuable to explain the pathogenesis of DEACMP [7]. However, there is a lack of evidence in this regard.

DNA methylation refers to the selective attachment of methyl groups to specific bases of a DNA molecule with the assistance of DNA methyltransferases. In general, DNA methylation downregulates gene expression by inhibiting transcription factor binding [8]. Notably, changes in DNA methylation and gene expression have profound effects on diverse neurodegenerative diseases. For instance, hypomethylation and high expression of the SNCA gene trigger Parkinson's disease through α‐synuclein aggregation and Lewy body formation [9]. Hypomethylation and overexpression of TREM2 are key contributors to Alzheimer's disease [10]. Hypermethylation and decreased expression of NR2B were related to the perioperative neurocognitive disorder [11]. Meanwhile, it has been reported that CO can elevate the methylation levels of Foxp3, IL4, IL10, and IFNγ in peripheral blood, and is associated with asthma, decreased diastolic blood pressure, and immune cell profile alterations [12, 13, 14]. Therefore, we hypothesized that CO may induce DNA methylation changes, thereby contributing to the occurrence and development of DEACMP by regulating the expression of relevant genes.

In this study, we determined DNA methylation and expression profiles from peripheral blood samples of healthy individuals (normal controls, NCs), of patients with acute carbon monoxide poisoning (ACMP), and of patients with DEACMP, to identify genes and their epigenetic alterations linked to the onset of DEACMP. Subsequently, we conducted pyrosequencing to assess the robustness of the epigenetic markers identified. To determine if similar alterations occur in the brain, we validated our findings using brain tissue from a rat model for DEACMP. This study aimed to identify genes potentially involved in the pathogenesis of DEACMP by analyzing epigenetic gene modifications and expression regulation. The goal was to enhance the understanding of the role of DNA methylation modifications in DEACMP pathogenesis and to identify epigenetic biomarkers and potential targets for intervention.

## 2 Methods

### 2.1 Specimen Source

#### 2.1.1 Clinical Samples

A total of 48 individuals were enrolled in this study, including 32 patients who visited the emergency department of the First Affiliated Hospital of Zhengzhou University due to ACMP or DEACMP between December 2020 and March 2022, and 16 healthy individuals from the Physical Examination Department. Based on previously established inclusion and exclusion criteria, patients were divided into an ACMP and a DEACMP group [15, 16]. This study included both male and female participants to minimize potential confounding effects of sex, despite evidence suggesting that sex may not be associated with the onset of DEACMP [17]. This study was approved by the Ethics Committee of the First Affiliated Hospital of Zhengzhou University (2019‐KY‐190).

#### 2.1.2 Animal Samples

In consideration of the significance of sex as a biological variable (SABV), this study incorporated 80 Sprague–Dawley rats with a 1:1 ratio of males to females (6–8 weeks old weighing 200–250 g) to ensure that the findings were robust, generalizable, and reflective of biological variability. These rats were obtained from the Experimental Animal Center of Zhengzhou University and maintained on a 12‐h light–dark cycle with free access to food and water. All experimental procedures were approved by the Animal Ethics Committee of Zhengzhou University (ZZU‐LAC20240322 [25]). The DEACMP model was established as described by previous researcher [18]. The rats were euthanized by intraperitoneal injection of 100 mg/kg pentobarbital sodium, and hippocampal tissue was collected and stored at −80°C for subsequent experiments.

### 2.2 DNA Methylation Sequencing and DMPs

The OMEGA Tissue DNA Kit and EZ DNA Methylation Gold Kit (Zymo Research, Irvine, CA, USA) were used for DNA extraction and bisulfite conversion, respectively, according to the manufacturer's instructions. The DNA methylation profiles of peripheral blood samples were analyzed using a Methylation 850 K BeadChip (Illumina, San Diego, CA, USA) from the Shanghai Biotechnology Corporation. Raw methylation data were processed using the “minifi” package and normalized using the subset‐quantile within array normalization (SWAN) method. Probes were excluded if they had a *p* value ≥ 0.01 in at least one sample. Methylation levels are denoted as *β*‐values, ranging from zero (unmethylated) to one (fully methylated). Consistent with a previous study, differential methylation analysis was performed using the “limma” package [19]. Differences in mean *β*‐value (Δβ) for each CpG site between the two groups were calculated. CpGs sites with an absolute Δ*β* value ≥ 0.1 and *p* < 0.05 were considered to be differentially methylated.

### 2.3 Pyrophosphate Sequencing

DNA extraction and bisulfite conversion were performed as described in the previous subsection. Primers were designed using the PyroMark Assay Design software, version 2.0. For cg14021373, the primers sequences were as follows: forward, 5′‐GTGGGGAGGATAAAGATGTATT‐3′; reverse, 5′‐ATTCACCATATTAACCAAACTAATCTCTA‐3′; and sequencing, 5′‐TTGTATTTAATTTGTATTTTTTAGG‐3′. For cg04305804: forward, 5′‐AGAGGGTTGGTATTGATAATTAGTGA‐3′; reverse, 5′‐CAAATAATAAAAAACACCCAACCTATCA‐3′; and sequencing, 5′‐TCATTTTCTTTCTAAAATAATACT‐3′. Polymerase chain reaction (PCR) was performed using the TaKaRa EpiTaq HS (TaKaRa, Shiga, Japan) with the following conditions: 95°C for 5 min, followed by 40 cycles of 95°C for 30 s, 55°C for 30 s, and 72°C for 30 s, then 72°C for 5 min, and a hold at 4°C. Finally, the products were sequenced using PyroMark Q48 Autoprep (Qiagen, Germantown, MD, USA) to detect methylation levels.

### 2.4 RNA Sequencing and Differentially Expressed Gene (DEG)

The PX Blood RNA Kit (Cat#R1057‐02, OMEGA, Georgia, US) was used to extract RNA according to the manufacturer's instructions. RNA integrity was assessed using an Agilent 4200 TapeStation (Agilent Technologies, Santa Clara, CA, US). Total whole blood RNA was purified using the RNAClean XP Kit (Cat A63987; Beckman Coulter Inc., Brea, CA, USA) and an RNase‐Free DNase Set (Cat#79254, QIAGEN, GmBH, Germany). The RNA was processed and converted into complementary DNA (cDNA) and final sequence‐ready libraries using the VAHTS Universal V6 RNA‐seq Library Prep Kit for Illumina. Sequencing was performed using a NovaSeq6000 (Illumina). The raw reads had > Q20 base quality scores, indicating an error rate of less than 1 per 100 bp. After quality control and removal of rRNA reads, the processed reads were mapped to the human genome (GRCh38) using the spliced mapping algorithm implemented in Hisat2 (version 2.0.4). Differential expression analysis was performed on the raw read counts using the DESeq2 R package. Genes with *p* < 0.05 were considered to be differentially expressed.

### 2.5 Water Maze Experiment

The Morris water maze test was used to assess the learning and memory abilities of the experimental rats. After five consecutive days of training, rats unable to locate the platform in all quadrants were excluded from subsequent experiments. The remaining rats were exposed to carbon monoxide, and the surviving rats were tested in the water maze, with latency to locate the platform being recorded.

### 2.6 Hematoxylin–Eosin (HE) Staining

Hippocampal samples were fixed, embedded in paraffin, and sliced. Slides were de‐waxed in xylene, anhydrous ethanol, and 75% ethanol. Sections were prepared for hematoxylin and eosin staining after being washed with distilled water. Neuronal damage in the hippocampus was observed under a microscope (400× magnification) and scored based on the number of necrotic neurons [20].

### 2.7 Nissl Staining

Slides were de‐waxed using the same procedure as described in the previous subsection. The sections were then treated with Nissl dye for 5 min and with xylene for 10 min. The slides were sealed and observed under a microscope (400× magnification). Nissl‐positive cells were counted using ImageJ software.

### 2.8 TdT‐Mediated dUTP Nick‐End Labeling

TdT‐mediated dUTP nick‐end labeling (TUNEL) staining was employed to assess apoptosis of hippocampal neurons. Slides were de‐waxed using the procedure already described and washed with distilled water. The One‐Step TUNEL Apoptosis Assay Kit (Beyotime, Shanghai, China) was used. Briefly, paraffin slides were pretreated with proteinase K for 30 min and then treated with the TUNEL staining solution for 1 h. 4′,6‐diamidino‐2‐phenylindole (DAPI) was used to stain cell nuclei. Apoptosis was observed using an orthogonal confocal fluorescence microscope (ZEISS). Quantitative analysis was performed by counting the number of TUNEL‐positive cells under a 200× field of view using ImageJ software.

### 2.9 Immunohistochemistry

Paraffin sections were sequentially de‐waxed with xylene and anhydrous ethanol, treated with a 3% hydrogen peroxide solution for 25 min to inhibit endogenous peroxidase activity, and blocked with 3% BSA for 30 min. The sections were incubated overnight at 4°C with the primary antibody (DAB2IP, 1:200; SMYD3, 1:200). Slides were washed with PBS and incubated with horseradish peroxidase‐labeled goat anti‐rabbit IgG (1:200) for 50 min at room temperature, followed by 3,3′‐diaminobenzidine (DAB) substrate and hematoxylin staining. Results were observed under a light microscope (200×), and staining was scored automatically using the IHC Profiler plug‐in in ImageJ software with the following outcomes: highly positive (3), positive (2), low positive (1), and negative (0) [21]. All antibodies used for immunohistochemistry and western blotting were purchased from Proteintech (Wuhan, China; DAB2IP, 23,582‐1‐AP; SMYD3, 12,011‐1‐AP).

### 2.10 Western Blotting

Hippocampal tissues were treated with radioimmunoprecipitation assay (RIPA) lysing buffer and protease inhibitors, and ground into homogenates. After centrifugation, protein concentration was determined using a BCA kit (Thermo Fisher Scientific, Waltham, MA, USA). Proteins were separated using sodium dodecyl sulfate‐polyacrylamide gel electrophoresis (SDS‐PAGE) and transferred onto polyvinylidene fluoride (PVDF) membranes. After blocking with 5% skim milk, the PVDF membranes were incubated at 4°C overnight with primary antibodies against DAB2IP (1:8000), SMYD3 (1:750), and β‐actin (1:1000). The following day, the PVDF membranes were washed and incubated with horseradish peroxidase‐labeled goat anti‐rabbit IgG (1:5000). Images were captured using enhanced chemiluminescence (Thermo Fisher Scientific).

### 2.11 Quantitative Real‐Time Polymerase Chain Reaction

To detect mRNA levels of target genes, RNA was extracted from hippocampal tissues using TRIzol (Thermo Fisher Scientific). Following the manufacturer's instructions, RNA was converted to cDNA using the PrimeScript RT reagent Kit with gDNA Eraser (Takara, Japan). Next, PCR was performed using TB Green Premix Ex Taq II FAST qPCR (Takara). mRNA levels were quantified using the $2^{-\Delta \Delta C_{t}}$ method. Primer sequences are shown in Table S1.

### 2.12 Statistical Analysis

R 4.2.0 software was utilized to process, analyze, and visualize the data. Differences between categorical variables were identified using the chi‐squared test or Fisher's exact test. The Shapiro–Wilk test was used to determine if the distribution of the continuous variables satisfies normality. Student's *t*‐test or one‐way ANOVA was used for continuous variables that conformed to a normal distribution. Conversely, data that were not normally/Gaussian distributed were analyzed using nonparametric tests. Statistical significance was set at *p* < 0.05.

## 3 Results

### 3.1 Clinical Data Collection

This study included a discovery cohort (*n* = 18) and a validation cohort (*n* = 30). The clinical data for each group are presented in Tables 1 and 2. Age and sex were matched between the groups in the discovery and validation cohorts (*p* > 0.05). The discovery cohort consisted of four females and two males per group, with mean ages of 52.50 ± 7.06 years for the NC group, 51.00 ± 7.54 years for the ACMP group, and 52.33 ± 10.03 years for the DEACMP group. The validation cohort included three females and seven males per group, with mean ages of 56.30 ± 3.16 years for the NC group, 56.20 ± 8.04 years for the ACMP group, and 61.50 ± 11.95 years for the DEACMP group.

**TABLE 1:** Clinical data summary and statistical analysis of the discovery cohort.

	NC	ACMP	DEACMP	*p*
Total number	6	6	6	
Sex (%)				
Female	4 (66.7)	4 (66.7)	4 (66.7)	1
Male	2 (33.3)	2 (33.3)	2 (33.3)	
Age ± SD	52.50 ± 7.06	51.00 ± 7.54	52.33 ± 10.03	0.943

**TABLE 2:** Clinical data summary and statistical analysis of the validation cohort.

	NC	ACMP	DEACMP	*p*
Total number	10	10	10	
Sex (%)				
Female	3 (30.0)	3 (30.0)	3 (30.0)	1
Male	7 (70.0)	7 (70.0)	7 (70.0)	
Age ± SD	56.30 ± 3.16	56.20 ± 8.04	61.50 ± 11.95	0.298

### 3.2 DNA Methylation Landscape of DEACMP

**FIGURE 1:**
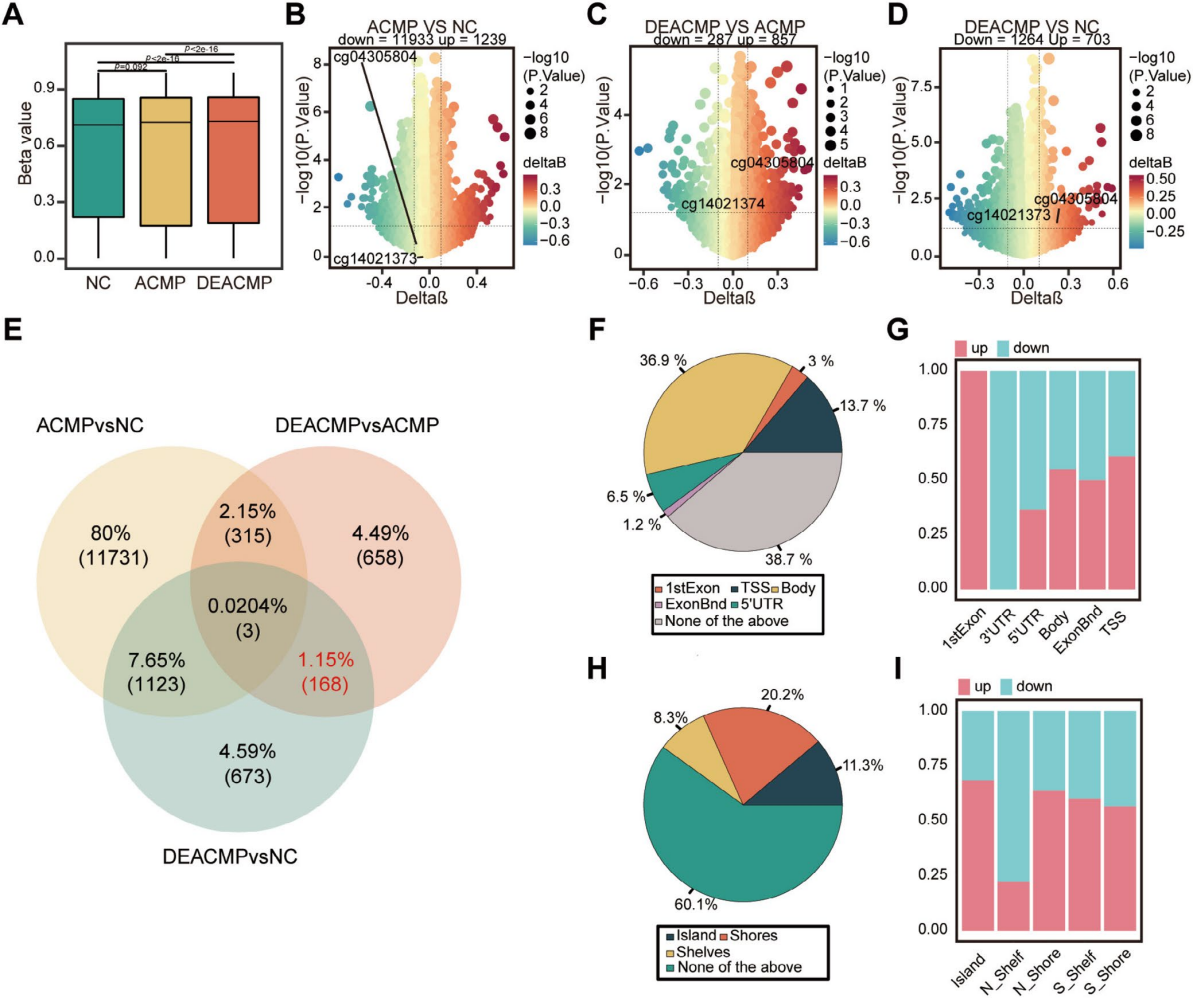
Differential methylation landscape of DEACMP at the global level and single site level. (A) Boxplot showing the overall methylation level of each group (Wilcoxon's rank sum test with Bonferroni's posttest, *n* = 6 per group). (B–D) Volcano plots depicting DMPs between the NC and ACMP groups, the NC and DEACMP groups, and the ACMP and DEACMP groups. (E) Venn diagram showing the intersection of DMPs between groups. (F, G) Genomic location distribution and up and downregulation ratios of DMPs. (H, I) Distribution of CpG island positions and up and downregulation ratios of DMPs. The data are presented as the mean ± SD. ACMP, acute carbon monoxide poisoning; DEACMP, delayed encephalopathy after carbon monoxide poisoning; DMP, differentially methylated point; NC, normal controls.

First, genome‐wide methylation analysis showed a significant upregulation of average methylation levels in the ACMP and DEACMP groups compared to the NC group (Figure 1A, *p* < 0.001). Remarkable variations were observed among the three groups (Figure 1B–D). Compared with the NC group, 1239 hypermethylated differentially methylated points (DMPs) and 11,933 hypomethylated DMPs were detected in the ACMP group. Additionally, 703 hypermethylated DMPs and 1264 hypomethylated DMPs were observed in the DEACMP group. Compared to the ACMP group, the DEACMP group contained 857 hypermethylated and 287 hypomethylated DMPs.

To characterize the specific alterations in DEACMP, we investigated the DMPs that differed between the DEACMP and ACMP groups, as well as the DMPs that highlighted the heterogeneity between the DEACMP and NC groups. We then selected the CpG sites with stable methylation levels between the ACMP and NC groups for further analysis, resulting in 168 DMPs linked to DEACMP (Figure 1E). The methylation patterns and annotations of the DMPs related to DEACMP are shown in Table S2.

Regional distribution analysis revealed that the majority of these DEACMP‐related DMPs were located in the body and promoter regions (Figure 1F), as well as in the shore and island regions (Figure 1H). Hypermethylated DMPs were enriched in the 1st exon, promoter, body, and island regions. Hypomethylated DMPs were mainly concentrated in the untranslated and shelf regions (Figure 1G,I).

In line with gene annotations, these DMPs were matched to 87 differentially methylated genes (DMGs). Fifteen DMGs were located in the promoter region, including eight hypomethylated genes (DAB2IP, PDE11A, ARPC3, OSCAR, TNFRSF18, REPIN1, NDST2, and C21of56) and seven hypermethylated genes (PM20D1, LIPN, CACNG3, C8of31, LOC101927526, PCDHGB3, and BANF2). These findings suggest that methylation changes in DEACMP primarily occur at the single‐CpG site level rather than at the overall level.

### 3.3 DEACMP‐Related Differentially Expressed Genes (DEGs)

**FIGURE 2:**
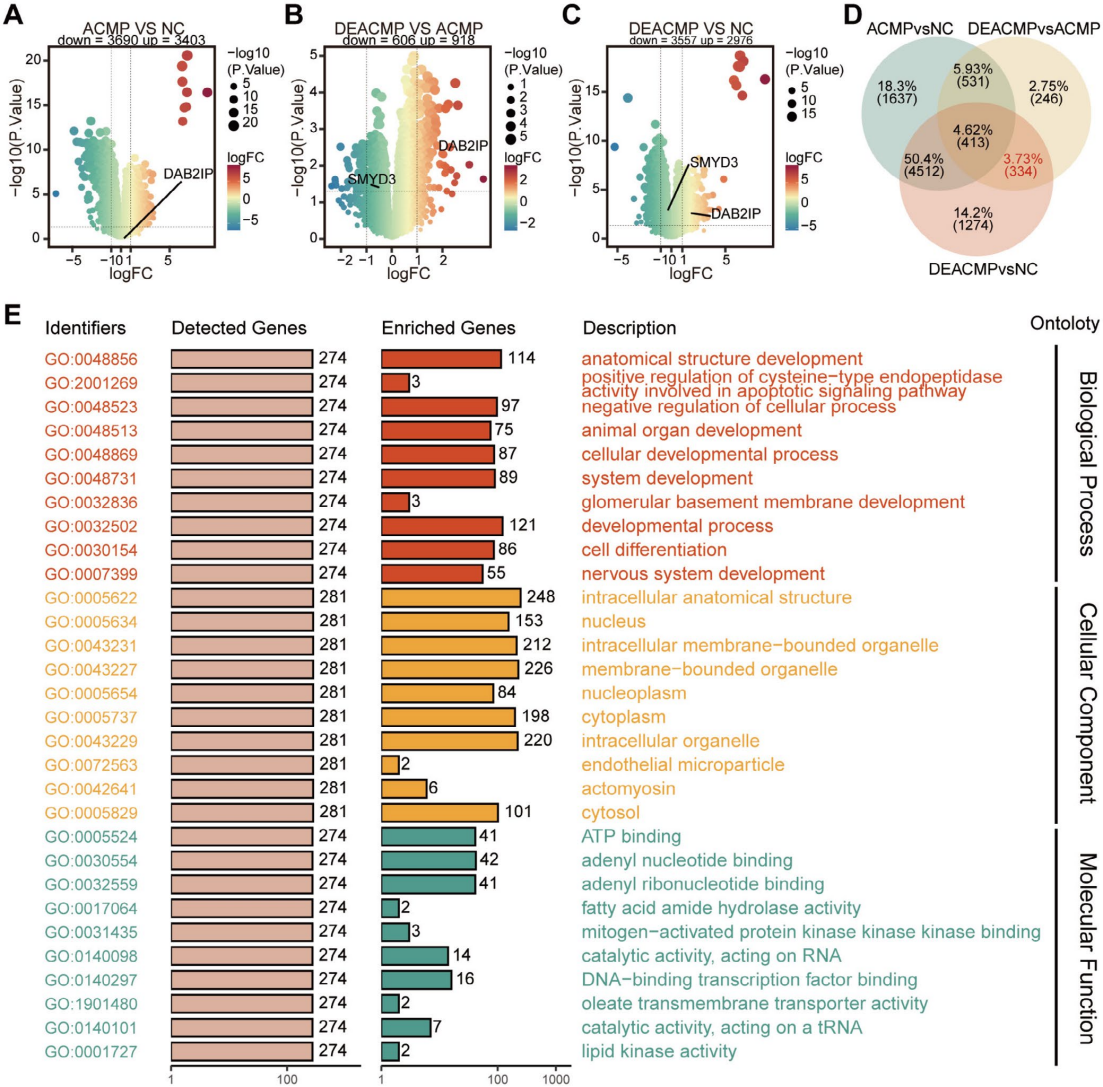
Screening of DEACMP‐related DEGs and gene ontology. (A–C) Volcano plots depicting DEGs between the NC and ACMP groups, the NC and DEACMP groups, and the ACMP and DEACMP groups. (D) Venn diagram showing the intersection of DEGs between groups. (E) Gene Ontology of DEACMP‐related DEGs. The data are presented as the mean ± SD. ACMP, acute carbon monoxide poisoning; DEACMP, delayed encephalopathy after carbon monoxide poisoning; DEG, differentially expressed gene; NC, normal controls.

To explore which gene transcription levels were altered in DEACMP, we performed a transcriptome‐wide analysis of the validation cohort. Compared to the NC group, we identified 3403 upregulated and 3690 downregulated genes in the ACMP group, whereas in the DEACMP group, the number of upregulated and downregulated genes was 2976 and 3557, respectively. Compared to the ACMP group, we identified 918 upregulated and 606 downregulated genes in the DEACMP group (Figure 2A–C).

Using the same strategy mentioned above for identifying DEACMP‐related DMPs, we identified 334 DEACMP‐related DEGs (Figure 2D). Gene ontology analysis indicated that these DEGs were associated with biological processes such as positive regulation of apoptotic signaling pathways, negative regulation of cellular processes, and nervous system development. Regarding cellular components, these DEGs were related to intracellular anatomical structures and to the nucleus. In terms of molecular function, these DEGs were related to adenyl nucleotides and DNA‐binding transcription factor binding (Figure 2E).

### 3.4 Integrated Analysis of the Methylome and Transcriptome

**FIGURE 3:**
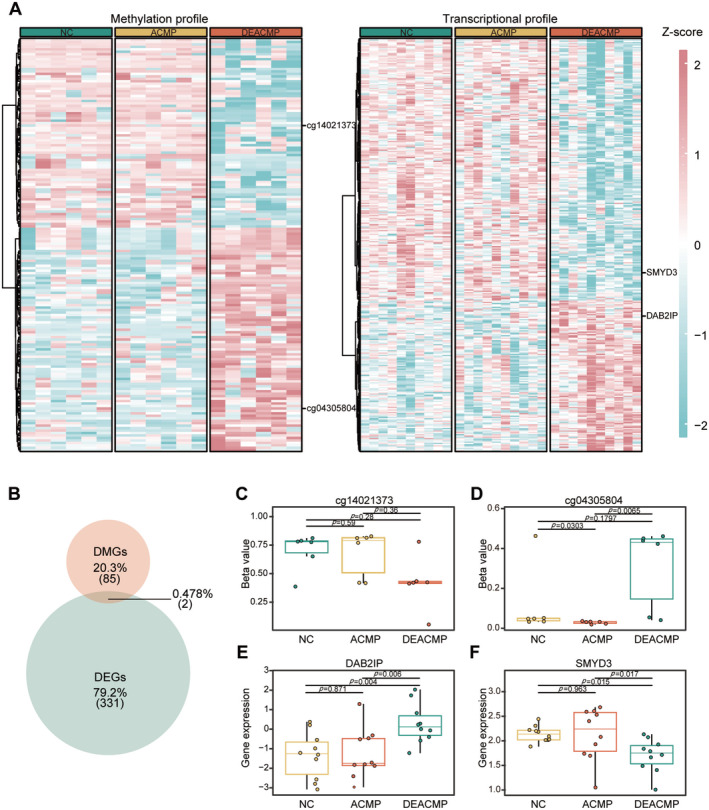
Integrative analysis of methylation profiles and transcriptome screening two DEACMP‐related specific genes. (A) Heatmap illustrating the methylation levels of DMGs and expression levels of DEGs. Beta values characterizing methylation levels and tpm values characterizing expression levels were subjected to Z‐score transformation. (B) Venn diagram showing the intersection of DEGs and DMGs. (C, D) Boxplots showing two CpGs, cg14021373 in the promoter region of DAB2IP and cg04305804 in the body region of SMYD3 with significantly altered methylation levels (Wilcoxon's rank sum test with Holm's posttest, *n* = 6 per group). (E, F) Boxplots showed two genes, DAB2IP and SMYD3 with significantly altered mRNA levels (One‐way ANOVA with LSD's posttest, *n* = 10 per group). The data are presented as the mean ± SD. ACMP, acute carbon monoxide poisoning; DEACMP, delayed encephalopathy after carbon monoxide poisoning; DEG, differentially expressed gene; DMG, differentially methylated gene; NC, normal control.

As previously mentioned, we screened 168 DEACMP‐related DMPs and 334 DEGs; their methylation and expression levels are shown in Figure 3A. To verify whether methylation changes in DEACMP led to dysregulated expression of related genes, we integrated methylome and transcriptome data and identified two epigenetic targets associated with DEACMP: cg14021373 in the promoter region of DAB2IP, and cg04305804 in the body region of SMYD3 (Figure 3B, Table S3).

cg14021373 showed a decreased methylation level while the expression of DAB2IP was upregulated (Figure 3C,E). Conversely, the methylation level of cg04305804 was elevated, whereas SMYD3 expression was downregulated (Figure 3D,F).

### 3.5 Pyrophosphate Sequencing Validated DEACMP‐Related Epigenetic Markers

**FIGURE 4:**
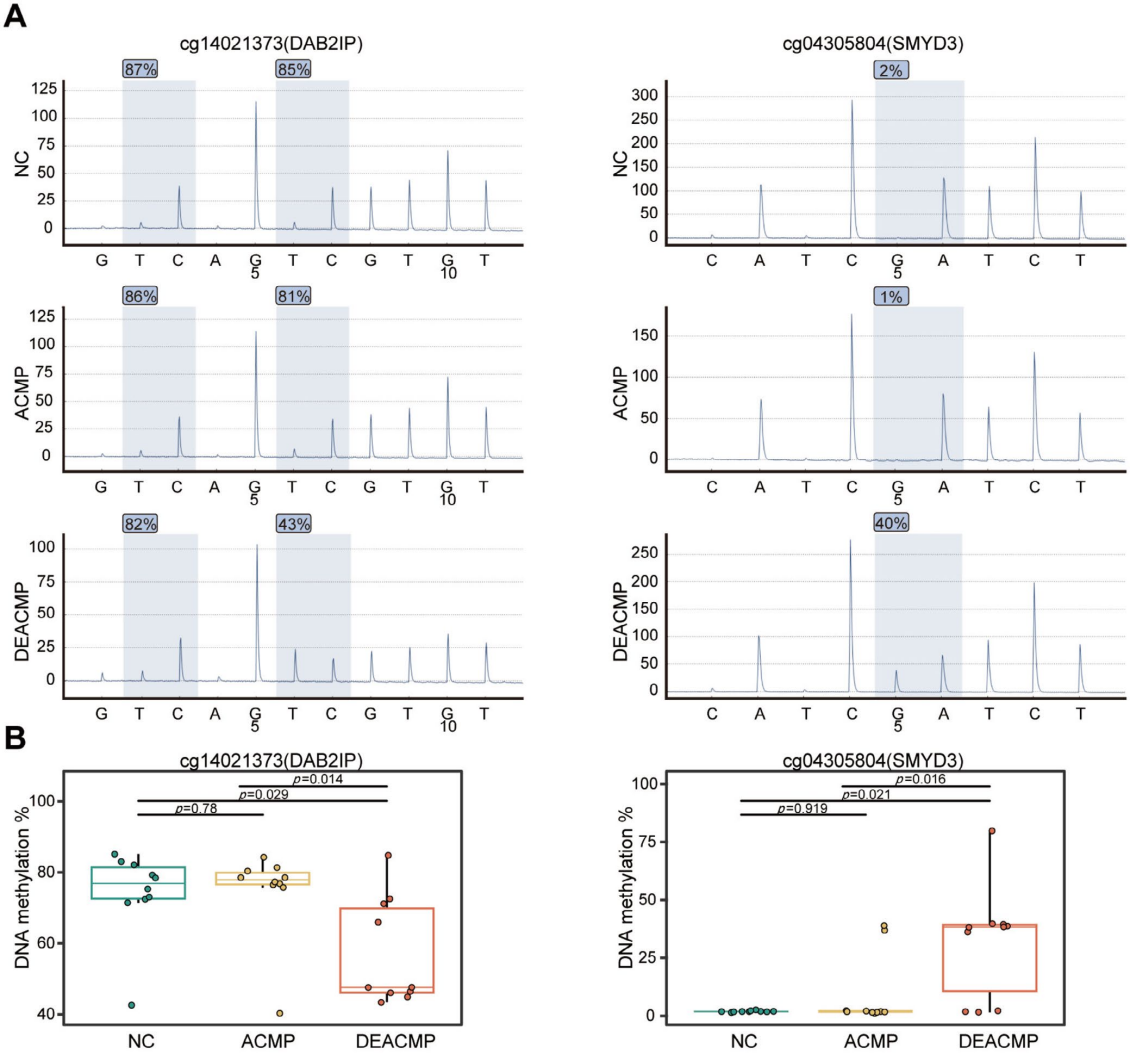
The epigenetic markers cg14021373 and 04305804 in peripheral blood of validation cohort were verified by pyrophosphate sequencing. (A) Representative pyrophosphate sequencing analysis of cg14021373 and 04305804 among the NC, ACMP, and DEACMP groups. (B) Boxplots showing the average DNA methylation levels of cg14021373 and 04305804 among NC, ACMP, and DEACMP groups (Wilcoxon's rank sum test with Bonferroni's posttest, *n* = 10 per group). The data are presented as the mean ± SD. ACMP, acute carbon monoxide poisoning; DEACMP, delayed encephalopathy after carbon monoxide poisoning; NC, normal control.

Pyrophosphate sequencing confirmed methylation changes of cg14021373 and cg04305804 in an expanded validation cohort. Compared to the NC group, methylation at cg14021373 showed minimal change in the ACMP group, but was significantly reduced in the DEACMP group (*p* < 0.05). Conversely, methylation at cg04305804 was significantly higher in the DEACMP group (Figure 4A). Despite variability in methylation levels within the DEACMP group, these two CpG sites consistently distinguished DEACMP cases (Figure 4B).

### 3.6 Delayed Cognitive Impairment and Brain Tissue Damage in Rats Induced by CO Exposure

**FIGURE 5:**
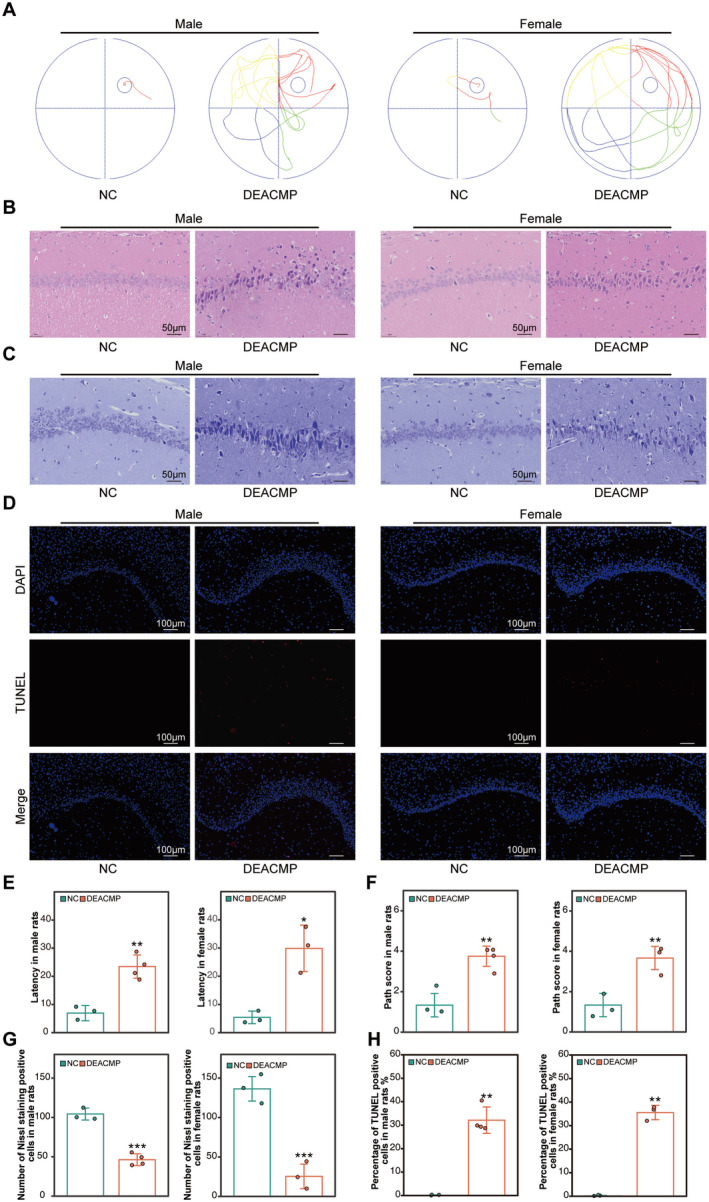
Cross‐sex robustness of behavioral and pathological in DEACMP rats. (A) Swimming trajectory plots of male and female rats in the Morris water maze test. (B) HE staining results of hippocampal tissues in male and female rats. (C) Nissl staining results of hippocampal tissues in male and female rats. (D) The TUNEL fluorescence staining results for male and female DEACMP rats. (E) Histogram indicating prolonged latency in the Morris water maze test for male and female DEACMP rats (*p < 0.05, **p < 0.01. Student's *t*‐test, *n* = 4 in male rats and *n* = 3 in female rats). (F) Histogram indicating increased histopathological scores in male and female DEACMP rats (**p < 0.01. Student's *t*‐test, *n* = 4 in male rats and *n* = 3 in female rats). (G) Histogram indicating reduced numbers of Nissl‐stained positive neurons in male and female DEACMP rats (***p < 0.001. Student's *t*‐test, *n* = 4 in male rats and *n* = 3 in female rats). (H) Histogram indicating the increased proportion of apoptotic cells in the hippocampus of male and female rats (**p < 0.01. Student's *t*‐test, *n* = 4 in male rats and *n* = 3 in female rats). The data are presented as the mean ± SD. DEACMP, delayed encephalopathy after carbon monoxide poisoning; NC, normal control.

In this study, we simultaneously incorporated male and female Sprague–Dawley rats and conducted stratified analyses to verify the cross‐gender robustness and reproducibility of the DEACMP biomarkers we identified. Following the methodology described by Thom [22] we established a rat model for DEACMP. When mortality rates were analyzed, male rats had a mortality rate of 75% (30/40), while females had a rate of 77.5% (31/40). The success of the DEACMP model was determined using water maze test and HE staining results (Figure 5A,B,E,F). The success rate was 10% (4/40) in male rats and 7.5% (3/40) in female rats. Both male and female DEACMP rats exhibited disorganized swimming trajectories and significantly prolonged latency compared to the NC group (Figure 5A,E). HE staining of hippocampal sections revealed notable pathological changes in the DEACMP group, including shrunken neurons, reduced cell size, vacuolation, irregular cell shapes, hyperchromatic nuclei, unclear nuclear‐cytoplasmic boundaries, widened intercellular spaces, and blurred layering (Figure 5B). Pathological scores were significantly higher in the DEACMP group compared to the NC group, confirming the severity of tissue damage (Figure 5F). Nissl staining and TUNEL assays further demonstrated hippocampal damage in DEACMP rats, characterized by reduced Nissl bodies, lighter cytoplasmic staining, significant neuronal damage, and an increased number of apoptotic neurons (Figure 5C,D,G,H). When comparing the behavioral and pathological differences between female and male DEACMP rats, the results were not statistically significant (*p* > 0.05). These findings provided further evidence that there was no difference in the mortality and success rate of DEACMP model between male and female rats, suggesting that the onset of DEACMP may not be sex‐dependent.

### 3.7 DAB2IP and SMYD3 Expression in DEACMP Rat Brain

**FIGURE 6:**
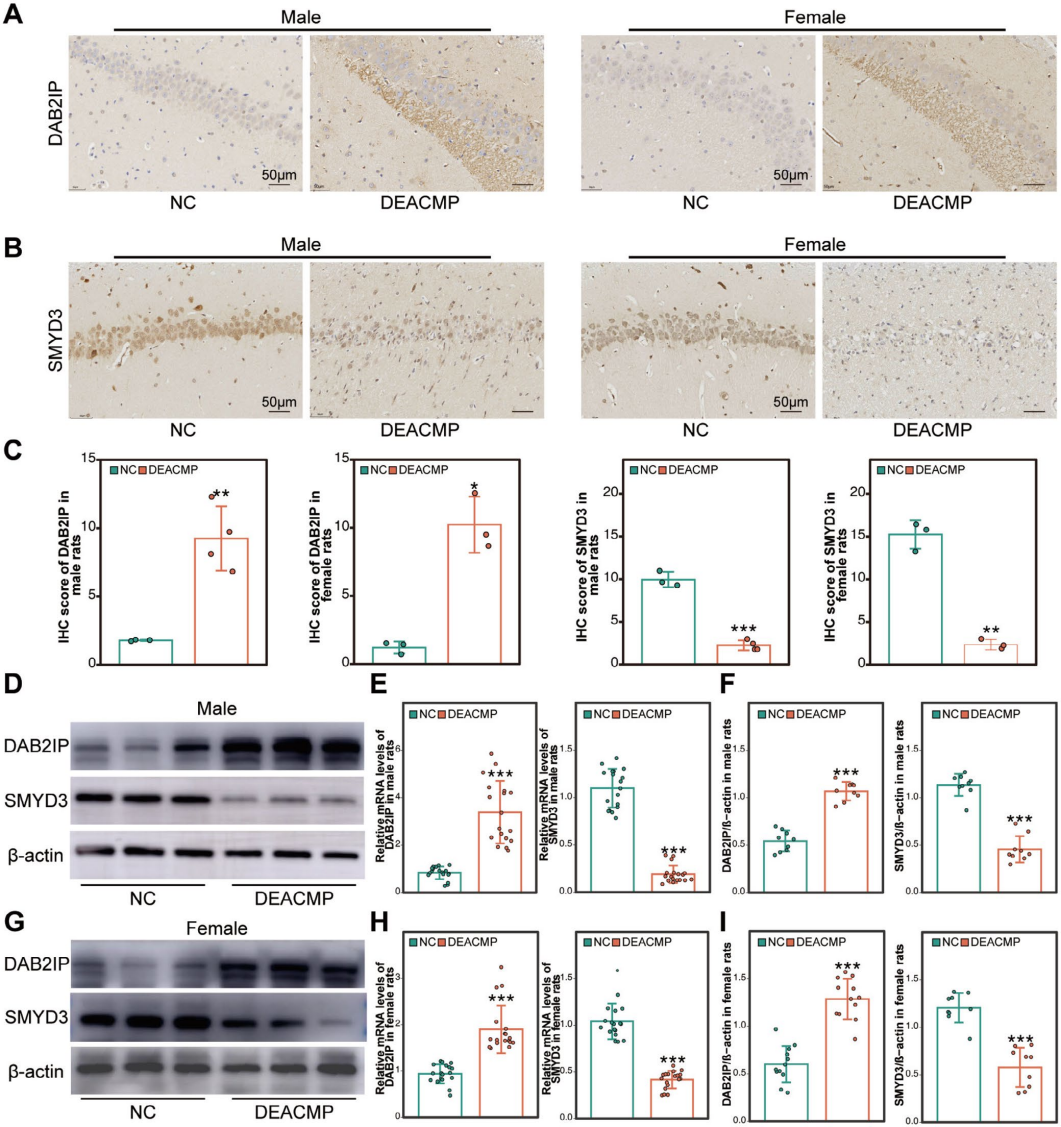
Cross‐sex robustness of DEACMP biomarker DAB2IP and SMYD3. (A, B) The immunohistochemical results of DAB2IP and SMYD3 in the hippocampus of female and male DEACMP rats. (C) Histogram indicating the semi‐quantitative analysis of the immunohistochemical results based on IHC profiler (*p < 0.05, **p < 0.01, ***p < 0.001. Student's *t*‐test, *n* = 4 in male rats and *n* = 3 in female rats). (D, G) Western blot of DAB2IP and SMYD3 in hippocampal tissues of male and female rats. (E, H) Comparison of DAB2IP and SMYD3 mRNA levels by qRT‐PCR analysis in hippocampal tissues of male and female rats (***p < 0.001. Student's *t*‐test, *n* = 18 per group). (F, I) Histogram indicating quantitative analysis of DAB2IP and SMYD3 protein levels in hippocampal tissues of male and female rats (***p < 0.001. Student's *t*‐test, *n* = 9 per group). The data are presented as the mean ± SD. DEACMP, delayed encephalopathy after carbon monoxide poisoning; NC, normal control.

Importantly, we also measured the expression levels of DEACMP candidate biomarkers DAB2IP and SMYD3 in both male and female DEACMP rat model, conducting three biological replicates. First, the immunohistochemistry staining revealed that compared to the NC group, the cytoplasm of hippocampal neurons in male and female DEACMP rats exhibited stronger DAB2IP staining, while the staining of SMYD3 in the nucleus was weaker. Quantitative analysis showed that these differences were statistically significant (Figure 6A–C). Meanwhile, qRT‐PCR results demonstrated a significant increase in DAB2IP mRNA levels and a notable decrease in SMYD3 mRNA levels in the hippocampus of DEACMP rats, consistent with the transcriptomic data (Figure 6E,H). Additionally, at the protein level, DAB2IP expression increased while SMYD3 expression decreased in hippocampal tissues (Figure 6D,F,G,I). These findings demonstrated consistent expression patterns of the DEACMP‐related genes SMYD3 and DAB2IP in both brain tissue from experimental animals and blood samples from clinical patients. Meanwhile, they denoted the cross‐gender robustness and reproducibility of DAB2IP and SMYD3 as potential DEACMP biomarkers.

## 4 Discussion

DNA methylation modifications and their consequent gene expression changes are significant in various neurodegenerative diseases. However, the methylation and expression landscape specific to DEACMP remains unclear. Ethical considerations and clinical constraints make it challenging to obtain brain tissue samples from patients affected by DEACMP. Nevertheless, peripheral blood samples offer insights into DNA methylation and transcription levels to some extent [23, 24]. Liquid biopsy techniques based on peripheral blood are cost‐effective, minimally invasive, and easily collected. Therefore, in our study, we integrated patient peripheral blood methylome and transcriptome data from patients.

Initially, our study revealed an overall upregulation in the DNA methylation profile in DEACMP, identifying 168 DEACMP‐related CpG sites. Transcriptome analysis further identified 334 DEACMP‐related genes. Through data integration and biological function analysis, we identified two genes potentially involved in the pathology of DEACMP, namely DAB2IP and SMYD3, along with their methylation modifications. Subsequently, we validated the changes in DNA methylation, mRNA levels, and protein levels of these two genes through peripheral blood samples from patients with DEACMP and brain tissue samples from a rat model for DEACMP.

Extensive dysregulation of DNA methylation and gene expression is implicated in DEACMP development. In epigenetic studies, the focus typically centers on methylation changes in CpG islands within gene promoter regions, where promoter methylation can inhibit transcription factor binding, thereby silencing gene expression [25]. However, emerging evidence suggests that DNA methylation changes also occur in the gene body, shore, and shelf regions [26]. In our study, DMPs were predominately observed in these regions, similar to previous findings reported in cases of meningitis induced by *Glaesserella parasuis* [27]. Methylation changes in the gene body region have been linked to alternative exon splicing, while methylation alterations in the shore/shelf regions may affect gene expression via alternative transcription start sites [27]. Thus, variations in DNA methylation across different genomic locations play diverse regulatory roles in transcriptomic changes.

Our transcriptome analysis identified 334 DEACMP‐related DEGs. Functional analysis revealed their involvement in biological processes such as positive regulation of apoptotic signaling pathways, negative regulation of cellular processes, cell differentiation, and nervous system development. Consistent with these findings, a previous study has confirmed increased hippocampal neuronal apoptosis in DEACMP rats [28]. Additionally, Ochi et al. reported a reduced number of neural progenitor cells in the hippocampus, and dysregulated neurogenic factors, in this model, underscoring the disruption of adult hippocampal neurogenesis [29]. Molecular function analysis of the 334 DEACMP‐related genes revealed enrichment in functions such as adenyl nucleotide binding and DNA‐binding transcription factor binding. This aligns with findings by Nanavaty et al., who suggested that DNA methylation changes in promoter region can modulate gene expression by inhibiting transcription factor binding, while nonpromoter DNA methylation may influence gene expression through mRNA polyadenylation regulation [30], thereby linking dysregulated gene expression to DNA methylation modifications.

Genes regulated by DNA methylation warrant further investigation [9]. Initially, based on methylome and pyrosequencing data, we identified cg14021373 in the promoter region of DAB2IP and cg04305804 in the body region of SMYD3 as epigenetic markers in peripheral blood from patients with DEACMP. Interestingly, the trends in DAB2IP and SMYD3 expression were inversely correlated with their DNA methylation patterns. DAB2IP exhibited hypomethylation and increased expression, known to be associated with upregulated endoplasmic reticulum stress, which has been implicated in Alzheimer's disease, Parkinson's disease, and amyotrophic lateral sclerosis [31, 32, 33]. During endoplasmic reticulum stress, DAB2IP promotes the dimerization and phosphorylation of IRE1, subsequently triggering the activation of the ASK1‐JNK pathway, ultimately leading to apoptosis [33, 34]. Therefore, the increased hippocampal neuronal apoptosis in the hippocampus of our rat model may be linked to elevated DAB2IP expression. Conversely, SMYD3 exhibited hypermethylation and reduced expression, impacting pro‐apoptotic factors (Bim, Bak, and Bax) and decreasing anti‐apoptotic factors (Bcl‐2 and Bcl‐xl) [35], potentially contributing to neuronal damage in the hippocampus related to DEACMP. SMYD3 is a unique epigenetic modifier that promotes macrophage polarization from M1 to M2 by methylating histone H3K4me3 [36]. The role of SMYD3 in macrophage polarization, particularly toward the M1 phenotype in the central nervous system, aligns with previous reports on microglia polarization toward the M1 phenotype in DEACMP [37]. These pieces of evidence position DAB2IP and SMYD3 as potential therapeutic targets for the treatment of DEACMP.

By integrating the methylome and transcriptome data, our study unveiled dysregulated DNA methylation and gene expression profiles in DEACMP, providing compelling evidence for the involvement of epigenetic mechanisms in its pathogenesis. Moreover, we identified and validated DAB2IP and SMYD3 as DEACMP‐related genes and potential therapeutic targets, highlighting their methylation modifications as potential biomarkers. This study opens new avenues for predicting and treating DEACMP. Of note, this study was conducted using both male and female DEACMP rat models. On one hand, this ensures that the results are robust, generalizable, and reflective of biological variability. On the other hand, it suggests that the occurrence of DEACMP may not be sex‐dependent. However, our study has some limitations. First, this study was unable to capture the dynamic changes in DNA methylation. Second, owing to anatomical differences, the sequencing results from the peripheral blood samples from patients do not fully reflect alterations in their brain tissues.

## 5 Conclusion

In conclusion, this study elucidates the dysregulated DNA methylation and gene expression profiles that characterize DEACMP, identifying DAB2IP and SMYD3 as key players in the process. These findings underscore the role of DNA methylation in DEACMP pathogenesis and propose potential epigenetic biomarkers as well as potential therapeutic targets for DEACMP. This research lays the foundation for future investigations into treatment strategies for DEACMP.

## Author Contributions

Hongyi Yan, Ding Yuan, and Yan Zhang performed major experiments in this study and wrote the manuscript. Pinpin Jiang, Linlin Hou, Yue Wu, Yapeng Zhang, and Linshaung Zhao were involved in some experimental technical support. Yue Cheng, Fang Yang, Yuqi Du, and Huanzhou Zhu were involved in collecting clinical samples and clinical data information. Yi Li, Haihua Luo, Yong Jiang, and Yanxia Gao directed the project. Hongyi Yan, Yong Jiang, and Yanxia Gao verified the underlying data. All authors read and approved the final manuscript.

## Conflicts of Interest

The authors declare no conflicts of interest.

## Supporting information


Data S1.

Table S1.

Table S2.

Table S3.

## Data Availability

The data supporting the findings of this study are available from the corresponding author upon reasonable request.
