# Supplementary figures and images for: Integration of Methylation and Gene Expression Deciphered Candidate Biomarkers DAB2IP and SMYD3 in Delayed Encephalopathy After Carbon Monoxide Poisoning
Source: CNS Neurosci Ther. 2025 Feb 15;31(2):e70270. doi: 10.1111/cns.70270 (PMC11829117; doi:10.1111/cns.70270)

SMYD3

45kDa

65kDa

54kDa

42kDa

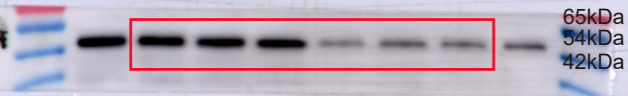

Supplement: Supplementary file 1 — Data S1. [file CNS-31-e70270-s001.zip › CNS70270-sup-0001-Full unedited (2).pdf]

$\beta$ -actin

45kDa

65kDa  
54kDa  
42kDa

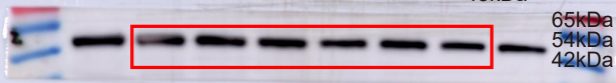

Supplement: Supplementary file 1 — Data S1. [file CNS-31-e70270-s001.zip › CNS70270-sup-0001-Full unedited (3).pdf]

# DAB2IP

118kDa

190kDa  
140kDa  
95kDa

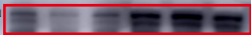

Supplement: Supplementary file 1 — Data S1. [file CNS-31-e70270-s001.zip › CNS70270-sup-0001-Full unedited (4).pdf]

SMYD3

45kDa

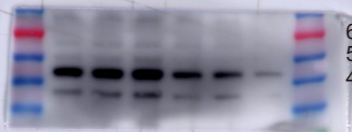

65kDa

54kDa

42kDa

Supplement: Supplementary file 1 — Data S1. [file CNS-31-e70270-s001.zip › CNS70270-sup-0001-Full unedited (5).pdf]

$\beta$ -actin

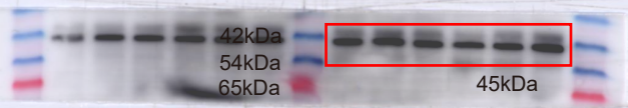

Supplement: Supplementary file 1 — Data S1. [file CNS-31-e70270-s001.zip › CNS70270-sup-0001-Full unedited (6).pdf]

DAB2IP

118kDa

190kDa  
140kDa  
95kDa

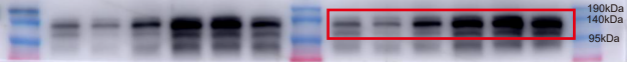

Supplement: Supplementary file 1 — Data S1. [file CNS-31-e70270-s001.zip › Full unedited gelblot for Figure (1).pdf]
